# Random Photon Absorption Model Elucidates How Early Gain Control in Fly Photoreceptors Arises from Quantal Sampling

**DOI:** 10.3389/fncom.2016.00061

**Published:** 2016-06-24

**Authors:** Zhuoyi Song, Yu Zhou, Mikko Juusola

**Affiliations:** ^1^Centre for Mathematics, Physics and Engineering in the Life Sciences and Experimental Biology (CoMPLEX), University College LondonLondon, UK; ^2^Department of Biomedical Science, University of SheffieldSheffield, UK; ^3^School of Engineering, College of Science and Technology, University of Central LancashirePreston, UK; ^4^State Key Laboratory of Cognitive Neuroscience and Learning, Beijing Normal UniversityBeijing, China

**Keywords:** photoreceptor, light adaptation, *Random Photon Absorption Model (RandPAM)*, sublinear summation, *quantum-gain-nonlinearity*, photon sampling, multi-photon-hits

## Abstract

Many diurnal photoreceptors encode vast real-world light changes effectively, but how this performance originates from photon sampling is unclear. A 4-module biophysically-realistic fly photoreceptor model, in which information capture is limited by the number of its sampling units (microvilli) and their photon-hit recovery time (refractoriness), can accurately simulate real recordings and their information content. However, sublinear summation in quantum bump production (*quantum-gain-nonlinearity*) may also cause adaptation by reducing the bump/photon gain when multiple photons hit the same microvillus simultaneously. Here, we use a *Random Photon Absorption Model* (*RandPAM*), which is the 1st module of the 4-module fly photoreceptor model, to quantify the contribution of *quantum-gain-nonlinearity* in light adaptation. We show how *quantum-gain-nonlinearity* already results from photon sampling alone. In the extreme case, when two or more simultaneous photon-hits reduce to a single sublinear value, *quantum-gain-nonlinearity* is preset before the phototransduction reactions adapt the quantum bump waveform. However, the contribution of *quantum-gain-nonlinearity* in light adaptation depends upon the likelihood of multi-photon-hits, which is strictly determined by the number of microvilli and light intensity. Specifically, its contribution to light-adaptation is marginal (≤ 1%) in fly photoreceptors with many thousands of microvilli, because the probability of simultaneous multi-photon-hits on any one microvillus is low even during daylight conditions. However, in cells with fewer sampling units, the impact of *quantum-gain-nonlinearity* increases with brightening light.

## Introduction

**Figure 1:**
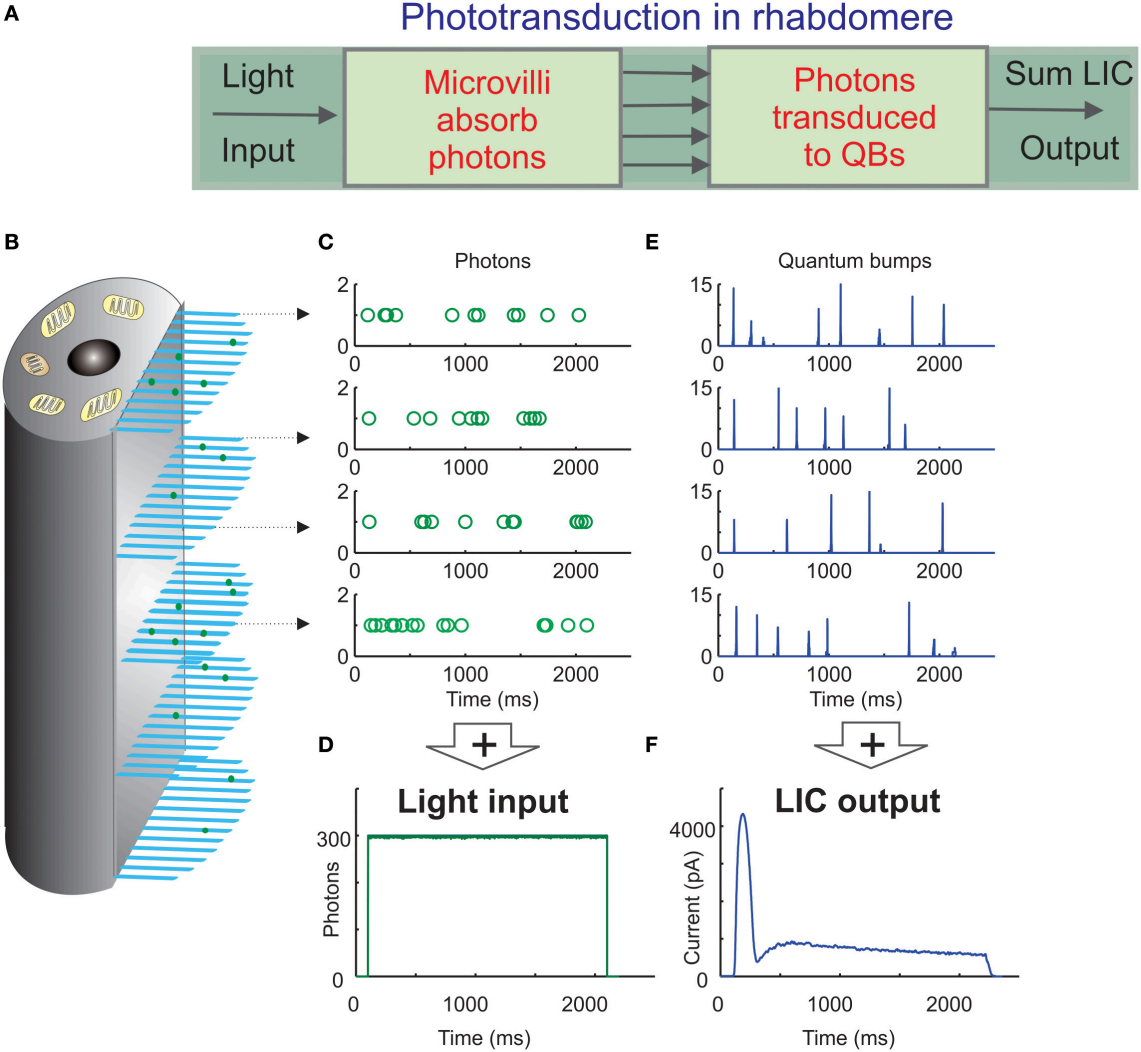
**Schematic of the biophysically realistic ***Drosophila*** photoreceptor model. (A)** The complete model's (Song et al., 2012) first three modules represent the phototransduction in the rhabdomere, which transduces light input (a dynamic flux of photons) into macroscopic output, light-induced current (LIC). **(B)** The rhabdomere contains 30,000 photon sampling units, microvilli (blue bristles). Each microvillus contains full phototransduction cascade reactions, and can transduce single photon (green dots) energies into unitary responses, quantum bumps (QB) of variable amplitudes and latencies. **(C)** In the 1st module, photons are randomly distributed over 30,000 microvilli (each row of open circles indicate a photon sequence absorbed by a single microvillus over time). **(D)** The light input (green trace) can be reconstructed by adding up all the photons distributed across the microvilli. **(E)** In the 2nd module, the successfully absorbed photons in each microvillus are transduced into QBs (a row of QB events). In each microvillus, the success of transducing a photon into a QB depends upon the refractoriness of its phototransduction reactions. The photons hitting a refractory microvillus cannot evoke QBs, but will be lost. This means that a microvillus cannot respond to the next photons until its phototransduction reactions have recovered from the previous photon absorption, which takes about 50–300 ms. **(F)** In the 3rd module, QBs from all the microvilli integrate the dynamic macroscopic LIC.

Fly photoreceptors can sample light changes across a truly astronomical input range—from a few photons in nightly shadows to billions in direct sunlight (Van Hateren, 1997)—and adapt this information in their limited (40–60 mV) output range. Although mechanistic understanding of photoreceptor adaptation is still incomplete (Hardie and Postma, 2008), recent research has elucidated its fundamental framework. Here, the biophysical fly photoreceptor model, which successfully uses stochastic sampling rules for simulating responses of real cells to a vast range of light stimuli (Song et al., 2012; Song and Juusola, 2014), has been influential. It utilizes a bottom-up approach to integrate signals from biophysically realistic sub-modules. Akin to a real fly photoreceptor, this design incorporates many thousands of independent photon sampling units (microvilli), which jointly act as the cell's photo-sensitive light-guide, the rhabdomere (Blue part in Figure 1B; Boschek, 1971).

Experiments indicate that each microvillus houses a full set of phototransduction reactants, from the rhodopsin molecules to the light-gated ion channels (Hardie and Postma, 2008). Because phototransduction reactions are stochastic and compartmentalized in single microvilli, they convert unitary photon-hits into unitary bioelectric responses, Quantum Bumps (QB), with a non-zero probability. Such information sampling can be modeled as a two-step process. First, a microvillus samples the photon(s) hitting it (Figure 1C). Second, if its internal reactions progress successfully, the absorbed photon energies are transduced into QBs (Figure 1D) (Hecht et al., 1942; Fuortes and Yeandle, 1964; Howard et al., 1987; Henderson et al., 2000). Most notably, each QB leaves a microvillus refractory for 50–300 ms, during which it cannot respond to a new photon. Finally, the QBs, arising from all the microvilli in the rhabdomere, sum up the graded macroscopic Light Induced Current (LIC) (Dodge et al., 1968; Juusola et al., 1994; Juusola and Hardie, 2001), which, in turn, drives the photoreceptor's voltage response.

Simulations imply that two mechanisms largely govern a fly photoreceptor's light adaptation: (i) its sample rate (QB rate) saturates, as more microvilli become refractory; and (ii) its sample waveform (QB size) shrinks due to Ca^2+^-dependent feedback and reduced electromotive force as the cell depolarizes (Juusola and Hardie, 2001; Song et al., 2012). Our model predicts that in normal daylight each mechanism contributes about 50% (Song et al., 2012; Song and Juusola, 2014). Notably, these two modes of adaptation (i and ii) are distinct from that of an alternative explanation, the sublinear bump summation hypothesis, which was also introduced recently (Pumir et al., 2008).

The sublinear bump summation hypothesis states that when more than one photon hits the same microvillus at the same time, multiple rhodopsins can be activated, but the resultant QB will be smaller than the sum of those produced independently. This could reduce the QB/photon gain by several folds (Pumir et al., 2008). However, the problem is that the likelihood of simultaneous multi-photon-hits has not been quantified, and therefore, their contribution to light adaptation is unknown.

The main aim of this paper is to quantify the probabilities for two or more photons hitting the same microvillus at the same time, and to elucidate what these events would mean to gain control in light adaptation. We do this by using the *Random Photon Absorption Model* (*RandPAM*) for a fly photoreceptor. *RandPAM* constitutes the first module of the complete fly photoreceptor model (Song et al., 2012; Song and Juusola, 2014). The complete model simulates the QB outputs of 30,000 microvilli, which sum up realistic whole-cell responses to any light intensity time-series stimulus (Figure 1 and Appendix). This was only possible because *RandPAM* provided realistic photon sequence input to all the microvilli. Here, we give *RandPAM*'s underlying assumptions and describe its derivation in detail.

In this paper, *RandPAM* is used to analyze the momentary input-output gain across the microvilli population, by calculating their average quantum charge. This is defined as the ratio between the total output charge of all bumps and the total number of incoming photons. Importantly, this definition removes the temporal dynamics from the analysis. We show how gain control emerges from photon sampling alone when a given photoreceptor has a finite number of sampling units (microvilli). This means that multi-photon-hits and their sub-linear summation predetermine an elementary form of gain control, which exists even without a phototransduction cascade adapting the subsequent QB waveform.

*RandPAM* treats each microvillus as an individual photon-sampling unit, and employs a compound binomial process to describe how photons are absorbed by a given number of microvilli. This approach further allows us to vary parametrically the key structural constraints, such as the number of microvilli, and quantify their impact on light adaptation. We show that for photoreceptors with many microvilli, the probability of simultaneous multi-photon-hits is minute, and thus these coincidences affect input-output gain only marginally. A typical fly photoreceptor has tens of thousands of microvilli (Boschek, 1971), each of which rarely experiences simultaneous multi-photon-hits, even in bright daylight (≤ 1%). However, for a photoreceptor with significantly fewer sampling units, such as the stick insect (*Carausius morosus*) (Frolov et al., 2012), multi-photon-hit induced gain control could play a bigger role in light adaptation.

## Random photon absorption model (*randPAM*)

### Fly photoreceptor structure

**Figure 2:**
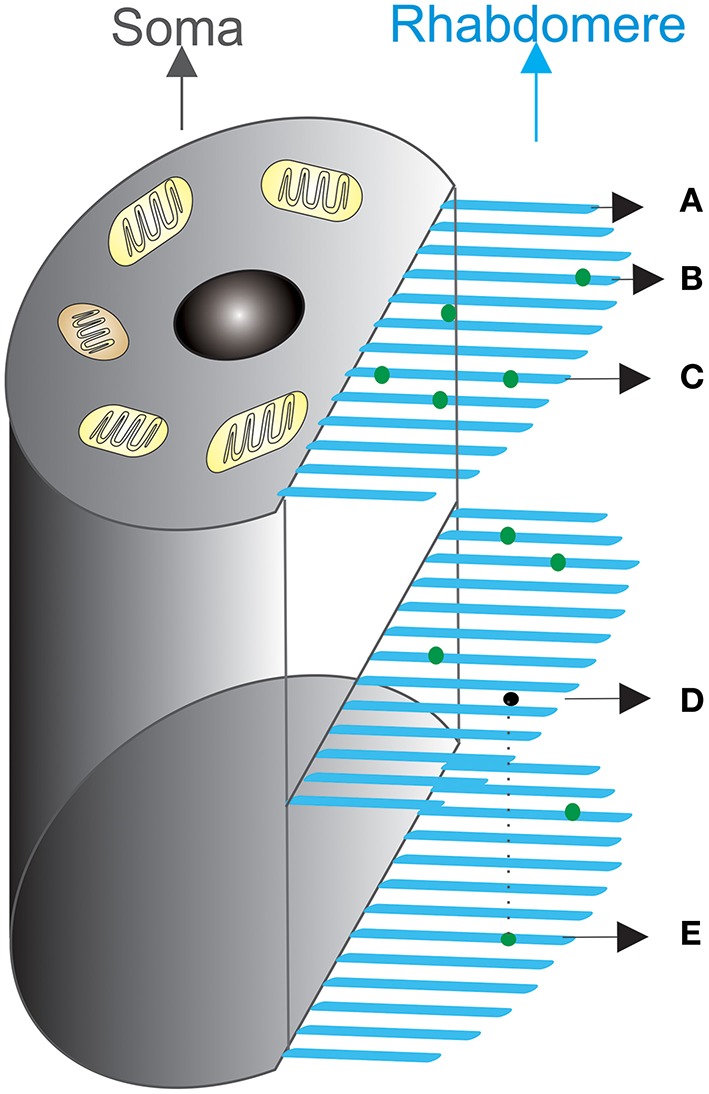
**Schematic of photon absorption process by a population of microvilli in a fly photoreceptor**. Microvilli (blue bristles) are stacked densely, layer by layer, at the side of the cell, forming the light-guide (rhabdomere). Light input at a time bin, Δ*t*, is the number of photons to be absorbed. These photons (green dots) enter the rhabdomere from above, propagate through its length, and are randomly distributed over 30,000 microvilli. Photons either hit a microvillus, being absorbed, or miss it, continuing to travel down to the next layers for possible absorption. **(A–E)** illustrates how photon absorption dynamics can differ at a particular time. **(A)** A microvillus that does not capture a photon; **(B)** A microvillus that captures one photon; **(C)** Two photons fall onto the same microvillus at the same time; **(D)** A photon can pass one microvillus layer, but **(E)** is captured at another layer. Light absorption statistics, which describe the probabilities of **(A–E)**, follow Poisson distribution, if the assumptions in Section Light Absorption by Poisson Process are made.

A rhabdomere (Figure 2) lies on a fly photoreceptor's apical surface. Each of its microvilli can be treated as an independent photon-sampling unit, and their collective photon absorptions define a photoreceptor's photon capture dynamics. Photons (dots) fall stochastically on to the stacked layers of microvilli (bars). A microvillus may thus be hit by no (A), one (B), two or multiple photons at once (C). Photons may also travel across one layer (D), but be captured at another (E).

Rhabdomeres of different species have different numbers of microvilli, which likely reflects each species' structural adaptations to different lifestyles and habitats. For example, the outer photoreceptors (R1–R6) of a dawn/dusk-active slow-flying fruit fly (*Drosophila melanogaster*) have 30,000 microvilli, whereas those of a midday-active fast-flying blowfly (*Caliphora vicina*) have 90,000 (Song et al., 2012). Theory and simulations suggest that a photoreceptor's total microvillus tally may constrain its signaling performance (Howard et al., 1987; Song et al., 2012; Song and Juusola, 2014).

In the next sections, we explore the structural limits of fly photoreceptors' encoding capabilities further. We ask: (i) whether an elementary form of gain control could directly result from the sampling process alone, (ii) and, how much can sublinear bump summation contribute to their light adaptation. But before we answer these questions, we define the underlying assumptions, and derive the relevant probabilities involved in photon sampling.

### Light absorption by poisson process

#### Assumptions

For deriving the light absorption statistics of a fly photoreceptor, the following assumptions were made explicitly:

1. *The input to the model is the number of photons absorbed by a photoreceptor*. For each light stimulus, we assume that 100% of its photons hit rhodopsin molecules, and these are converted to meta-rhodopsins. We further assume that the number of photons is fixed at a given intensity. This assumption is made specifically because we aim to quantify how the internal photon transductions constrain light adaptation, where the Poisson variations in the external light flux play no role.
2. *The light input only varies in intensity (quantified as photons/s)*. Although each photoreceptor type has its own specific photopigment, rhodopsin, with specific wavelength absorption probabilities that define its spectral sensitivity, assumption two is justified. Experiments have shown that each absorbed photon elicits a similar QB, irrespective of the energy that triggered it (Wu and Pak, 1975). This observation follows the principle of univariance (Rushton, 1972), here meaning that a photoreceptor's spectral sensitivity does not influence the QB waveform size, but only its absorption probability (Wu and Pak, 1975). Accordingly, the effective light intensity can be estimated with the specific photoreceptor type's spectral sensitivity function. Or, it can be gauged experimentally by counting QBs in dim light. From these counts, QB rates at brighter conditions can be extrapolated, without considering the wavelength, as we did in this study.
3. *All microvilli absorb photons independently*. In the rhabdomere, as light travels down the stacked microvillus array, slightly fewer photons should reach the bottom than the top layers (Figure 1B). Therefore, the bottom microvilli' photon hits depend upon the absorption of the layers above. For simplicity, this argument is ignored in the following calculations.
4. *All microvilli in the rhabdomere have the same photon absorption probability*. Microvillus lengths vary in the rhabdomere (Figure 1B). Microvilli shrink along the longitudinal axis and taper across the same cross-section, being longest in the middle. Presumably then, the smaller the microvillus, the smaller is its photon absorption probability. Thus, the top and middle microvilli' photon absorption probabilities are perhaps larger than those at the bottom or at the side (Stavenga, 2003). This assumption simplifies calculations by eliminating the role of geometry in the microvillar array.
5. *The number of photons absorbed within* one *microvillus at any time is independent of the past events*. This assumption follows the observation that the quantum bump occurrences seem random and independent (Fuortes and Yeandle, 1964; Henderson et al., 2000).

#### Derivation

Given the assumptions above, if we define a random variable *x* as the number of photons absorbed by one microvillus at one particular time-bin, *x* follows a binomial distribution:

(1) $$\begin{array}{cc} P(x)=\left(\begin{array}{l} N_{\text{ph}}\\ x \end{array}\right){\left(\frac{1}{N_{\text{u}}}\right)}^{x}\;{\left(1-\frac{1}{N_{\text{u}}}\right)}^{N_{\text{ph}}-x}, & \;(1) \end{array}$$

where *N*_u_ is the number of microvilli in the rhabdomere (in a *Drosophila* R1–R6 photoreceptor, *N*_u_ = 30,000), and *N*_ph_ is the number of photons in the light pulse at one time-bin, Δ*t*.

Equation (1) can be used to describe the photon absorption probability of a single microvillus. This is because the average probability that one photon hits a given microvillus is $\frac{1}{N_{\text{u}}}$, and that *x* photons hit the same microvillus is ${\left(\frac{1}{N_{\text{u}}}\right)}^{x}$. The probability that the other *N*_ph_ − *x* photons miss this microvillus is ${\left(1-\frac{1}{N_{\text{u}}}\right)}^{N_{\text{ph}}-x}$. In addition, there are $\left(\begin{array}{l} N_{\text{ph}}\\ x \end{array}\right)$ ways to select this set of *x* photons.

Subject to certain conditions, approximations could be made to *P*(*x*) (Bevington and Robinson, 2003). If *N*_ph_ and *N*_u_ are much larger than *x*, then:

(2) $$\begin{array}{cc} \begin{array}{l} P(x)\approx \frac{{\left(N_{\text{ph}}\right)}^{x}}{x!}{\left(\frac{1}{N_{\text{u}}}\right)}^{x}{\left(1-\frac{1}{N_{\text{u}}}\right)}^{N_{\text{ph}}}\\ \;\approx \frac{1}{x!}{\left(\frac{N_{\text{ph}}}{N_{\text{u}}}\right)}^{x}e^{-\frac{N_{\text{ph}}}{N_{\text{u}}}}\\ \;=\frac{{\left(\lambda_{\text{M}}\right)}^{x}e^{-\lambda_{\text{M}}}}{x!} \end{array}, & \;(2) \end{array}$$

where $\lambda_{\text{M}}=\frac{N_{\text{ph}}}{N_{\text{u}}}$ is the average number of photon-hits to each microvillus in the rhabdomere, assuming equal photon absorption probabilities for all microvilli (assumption 4). The result is a Poisson distribution, and the approximation in Equation (2) is valid if $x\ll \sqrt{N_{\text{ph}}}$, *x* ≪ *N*_u_, and 1 ≪ *N*_u_ are satisfied.

In practice, all approximations made in Equation (2) are valid near the region of *x* = λ_M_, if $N_{\text{ph}}\ll N_{\text{u}}^{2}$. In this situation, both $x=\lambda_{\text{M}}=\frac{N_{\text{ph}}}{N_{\text{u}}}\ll N_{\text{u}}$ and $x\ll \sqrt{N_{\text{ph}}}$ are satisfied. If *x* differs much from λ_M_, *P*(*x*) is essentially zero anyway. The condition $N_{\text{ph}}\ll N_{\text{u}}^{2}$ holds for most fly photoreceptors. For a typical *Drosophila* R1-R6 photoreceptor, it is well satisfied even at very bright intensities. Direct sunlight may carry 10^7−9^ photons/s to a photoreceptor's receptive field (Warrant and Mcintyre, 1992), but when using a reasonable integral time interval (less than 5 ms), the sampled number of photons is: $N_{\text{ph}}<5\times 10^{6}\ll N_{\text{u}}^{2}=9\times 10^{8}$.

For the ease of calculation, a random photon absorption model based on Poisson statistics is sometimes better than that based on binomial statistics, especially at dim light conditions ($N_{\text{ph}}<10^{3}$ photons/s). If the input were sampled at 1 kHz and each 1 ms were considered as one time-bin, the average number of photons absorbed by the photoreceptor at each time-bin would be less than one (*x* < 1). This situation would cause problems when calculating the absorption probabilities according to Equation (1).

#### Realization of random photon absorption model (randPAM)

In the section Derivation, a single microvillus is viewed as an independent photon-sampling unit. Following assumption (3) and (4), the random number of absorptions for different microvilli (*x*_*m*_, *m* = 1, 2…….*sN*_μ_) are independent, identically distributed Poisson random variables with mean $\lambda_{\text{M}}=\frac{N_{\text{ph}}}{N_{\text{u}}}$. However, there is an important physical constraint, which prevents us from calculating the number of absorbed photons for each microvillus with the Poisson distribution. Because we assume that the total number of incoming photons is a known fixed number (assumption 1) and that all the photons become absorbed, the photon-hits of all microvilli must add up to the input level, but not be higher ($Pro\left(\overset{N_{u}}{\underset{m=1}{\sum }}x_{m}>N_{ph}\right)=0$). This constraint would be violated if photon absorptions for all microvilli were realized through a Poisson distribution. Because the sum of independent Poisson variables still follows Poisson distribution, the mean of which equals to the sum of the individual means. This gives $\overset{N_{u}}{\underset{m=1}{\sum }}x_{m}\sim Poisson\left(N_{ph}\right)$, which means that the total number of absorbed photons may be higher than the total number of incoming photons ($\text{Pro}\left(\overset{N_{u}}{\underset{m=1}{\sum }}x_{m}>N_{ph}\right)>\;0$).

Here, we propose a practical way to solve this problem by considering the whole rhabdomere as a single unit. From Equation (1), one can calculate *P*(*x*), which is the probability of a single microvillus being hit by (i.e., it absorbs) *x* photons. The distribution that describes the number of microvilli that absorbs exactly *x* photons is again binomial, depending upon the value of *x*:

(3) $$\begin{array}{cc} Pro(y\;|\;x)=\left(\begin{array}{l} N_{\text{u}}\\ y \end{array}\right)P^{y}\;(x){\left(1-P(x)\right)}^{N_{\text{u}}\;-\;y}, & \;(3) \end{array}$$

where*y* is the number of microvilli that absorbs exactly *x* photons.

One could denote such a distribution as a compound binomial distribution: *y*|*x* ~ *B*(*N*_u_, *P*(*x*)), and *x* ~ *B*(*N*_ph_, 1/*N*_u_). Therefore, the expected number of microvilli that absorbs exactly *x* photons is *N*_u_*P*(*x*). This formula was previously used in Hochstrate and Hamdorf (1990) to calculate the number of activated microvilli for a blowfly photoreceptor in different light conditions. Here we defined the underlying assumptions and provided the corresponding derivations. And, we next use it to practically realize the stochastic photon absorption process and to decide how many photons each microvillus absorbs at one time-bin. The idea is to uniformly draw *N*_u_*P*(*x*) microvilli to absorb *x* photons, whereupon*x* varies from 1 to *x*_n_ (*x*_n_ is the maximum number that lets *N*_u_*P*(*x*_n_) > 1). In theory, if a random variable (x) were drawn from a Poisson distribution, x would not be limited by *x*_n_. However, in the simulations, this digitization limit exits: the minimum number of microvilli to absorb x photons is one.

An alternative (easier) way to compute the various probabilities of interest is by modeling the photon absorption process as a multinomial process. At each time incident, the distribution of *N*_*ph*_ photons over *N*_*u*_ microvilli is multinomial with size parameter equal to *N*_*ph*_, and probability vector of length *N*_*u*_ with each element equal to 1/*N*_*u*_.

#### Extension to continuous light condition

Assuming that photon-hits to a microvillus are independent across the time-bins, the extension to continuous light stimuli is straightforward. One can simply repeat the same light absorption process at each time-bin. However, the problem that still needs addressing is the sampling rate of the input stimuli; how long should the selected time-bin be?

As light intensity (input) increases, a photoreceptor's voltage response (output) becomes faster, utilizing a broader temporal frequency bandwidth. For slow-flying *Drosophila*, at bright light intensities at 25°C, the 3 dB cut-off frequency of its R1–R6 photoreceptors (the frequency at which the gain is the half maximum) is about 25 Hz. Accordingly, these cells respond little to light contrast changes above 100 Hz (Juusola and Hardie, 2001). Hence, by sampling light input at 200 Hz or above should adequately capture information in these photoreceptors' dynamical responses. But for a fast-flying killer fly (*Coenosia attenuata*), light input should be sampled minimally at 600 Hz to capture information in its photoreceptors' much quicker responses (Gonzalez-Bellido et al., 2011; Song et al., 2012). In this paper, light inputs were sampled at 1 kHz, making 1 ms as the standard time-bin.

### Estimating light input

When *RandPAM* is used to simulate real photoreceptor output, as we did in our previous publications (Song et al., 2012; Song and Juusola, 2014), careful calibration of the light input to *RandPAM* becomes very important. The light input to the *RandPAM* is the number of photons activating rhodopsin molecules per time bin.

The question is - how to estimate the total number of absorbed photons as precisely as possible? Considering the attenuating factors down the propagating light path, it is hard to estimate the number of absorbed photons from the photons emitted by the light source. Nevertheless, this number can be extrapolated more conveniently by using intracellular photoreceptor recordings. After prolonged dark-adaptation, QBs can be counted to continuous dim illumination at each second and used for extrapolating the photon-hit rates for brighter light levels (Laughlin and Lillywhite, 1982; Juusola et al., 1994; Juusola and Hardie, 2001). Evidence suggests that in dim conditions, the photon-to-QB relationship is statistically one-to-one, and that QBs increase linearly with light intensity, yet, this ratio saturates progressively at brighter intensities (Laughlin and Lillywhite, 1982; Juusola et al., 1994; Juusola and Hardie, 2001). Shot noise analyses of voltage responses in *Drosophila* R1-R6 photoreceptors also imply that the QB rate can be extrapolated linearly over dim and intermediate intensities, covering about 3 log units (Wu and Pak, 1978; Juusola and Hardie, 2001).

Although we use QB counts to determine the light input to the *RandPAM*, this method has its own limitations. The counts may include rare spontaneous bumps (< 1/min in a *Drosophila* R1-R6 photoreceptor Henderson et al., 2000). Furthermore, nonlinearities in photon absorption can be caused by the pupillary feedback control. Here, the intracellular pupil reduces the efficiency of photon-activating a rhodopsin molecule with brightening, as more photons are captured by the screening pigments (Vogt et al., 1982).

All these extrinsic and intrinsic changes in the absorption spectra and efficiency may bias the extrapolated photon rates. As this study assesses steady-state adapted photoreceptors, we can ignore nonlinear optical attenuation effects, such as intracellular pupil activation. Nevertheless, while the light intensity values (as extrapolated from the bump counts) may overestimate the photons-hits, these provide the best available and reasonably realistic photon rates (photons/s) for different light inputs, irrespective of the number of QBs they may evoke.

## Results

**Figure 3:**
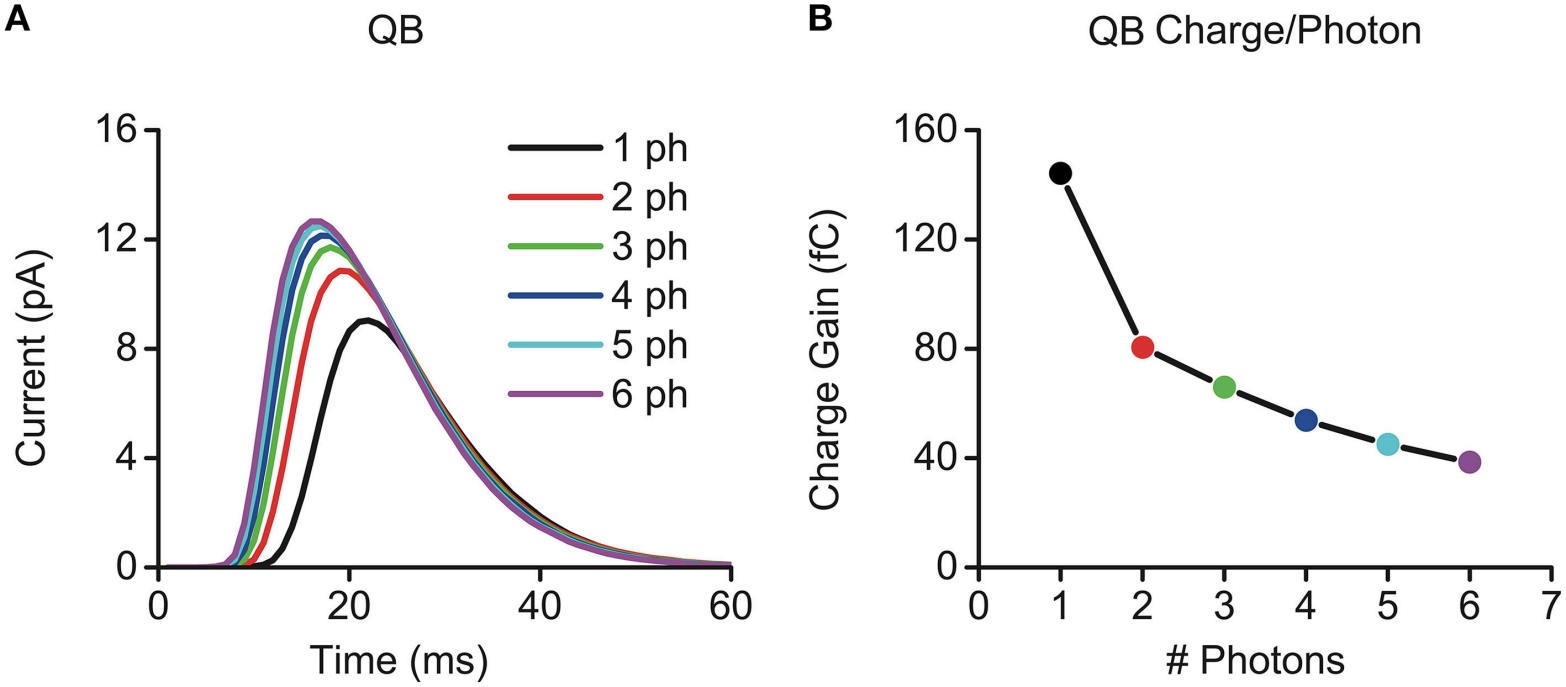
**Quantum-gain-nonlinearity induced by sublinear bump summation in multi-photon elicited QBs**. Here, quantum-gain-nonlinearity is illustrated through multi-photon evoked QBs, which were calculated by using the full *Drosophila* photoreceptor model, see Song et al. (2012). **(A)** Bigger QBs are evoked by multi-photon-hits. **(B)** The quantum gain (QB charge/photons) decreases nonlinearly. QB charge was calculated by integrating QB waveform in **(A)**, and its unit is fC (femto coulomb).

A microvillus can convert a single photon to a QB. But what happens when two or more photons hit it simultaneously? Computational studies imply that a bigger QB might be produced (Figure 3A), but the gain between the QB size and the number of photons would be reduced by several folds (Figure 3B) (Pumir et al., 2008; Song et al., 2012). In Figure 3B, we used the electric charge caused by a QB to define its size, which is the integral of a bump waveform. Because simultaneous multi-photon-hits are taken to induce sublinear summation in QB production, we define it here as *quantum-gain-nonlinearity*. In this section, we suggest how *quantum-gain-nonlinearity* may influence a photoreceptor's gain control in light adaptation.

We use the gain between the LIC output charge (*C*_out_) to the light intensity input (*L*_in_) to quantify a photoreceptor's input/output-relationship. For a discrete light pulse, *L*_in_ is composed of *N*_ph_ photons, and *C*_out_ is the summation of all excited QB charges. If we use *x* to denote simultaneous photon-hits to a single microvillus, and use $C_{x}^{QB}$ to denote the x-photon elicited QB charge in that microvillus, then, $C_{\text{out}}=\overset{x_{n}}{\underset{x=1}{\sum }}N_{x}C_{x}^{QB}$. Here *N*_*x*_ is the number of *x*-photons elicited QBs across the whole microvilli population, and *x*_n_ is the maximum number that lets *N*_u_*P*(*x*_n_) > 1. It then follows that:

(4) $$\begin{array}{cc} \frac{C_{\text{out}}}{L_{\text{in}}}=\frac{\sum_{x=1}^{x_{n}}N_{x}C_{x}^{QB}}{N_{\text{ph}}}. & \;(4) \end{array}$$

**Figure 4:**
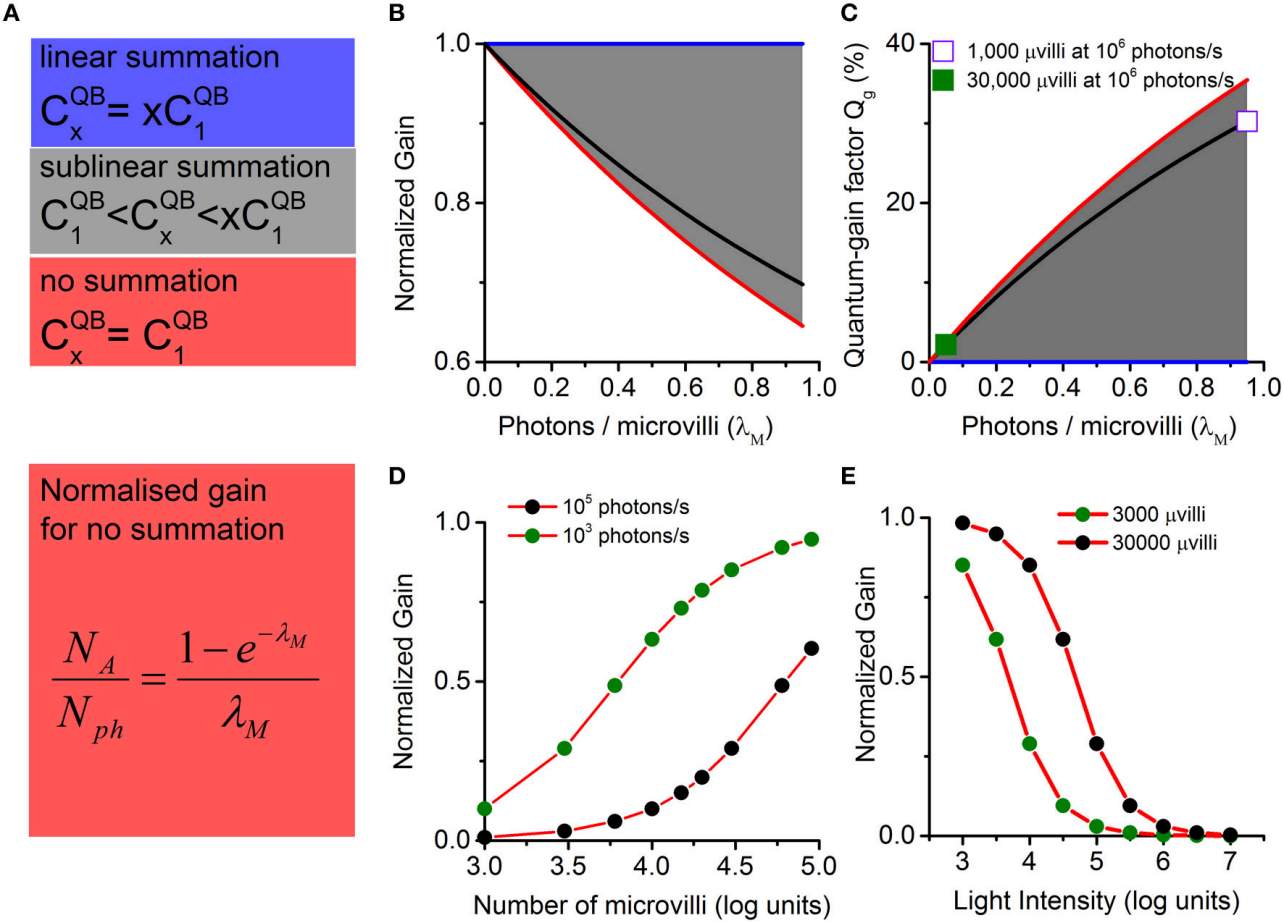
**Normalized gain and quantum-gain factor are bounded and change sublinearly with light intensity and number of microvilli**. The normalized gain is the actual gain divided by the linear gain ($\frac{C_{\text{out}}}{L_{in}C_{1}^{QB}}$). The quantum-gain factor is defined as the difference between the normalized linear gain and the normalized actual gain (1$-\frac{C_{\text{out}}}{L_{in}C_{1}^{QB}}$). **(A)** There are three cases for multi-photon-hits induced QB production: linear summation (blue), sub-linear summation (gray), and no summation (red). In the linear summation, x-photon induced bump charge ($C_{x}^{QB}$) is equal to x times a single photon induced bump charge ($C_{1}^{QB}$). The other extreme case is that no summation happens in QB production; thus, $C_{x}^{QB}$equals to $C_{1}^{QB}$. More often, sublinear summation occurs in QB production, where $C_{x}^{QB}$ is bounded between $C_{1}^{QB}$ and $xC_{1}^{QB}$. **(B)** Corresponding normalized gains (for the three cases in **A**). **(C)** Corresponding quantum-gain factors. Linear summation provides the upper bound for the normalized gain and the lower bound for the quantum-gain factor (blue lines in **B,C**). No summation provides the lower bound for the normalized gain and the upper bound for the quantum-gain factor (red lines in **B,C**). When sublinear summation occurs in QB production, normalized gain and quantum-gain factor fall in the gray areas in **(B,C)**. The black lines in **(B,C)** show a practical example, using $C_{x}^{QB}$ values calculated from Figure 3. Both the normalized gain and the quantum-gain factor change sublinearly with λ_M_. For example, the quantum-gain factor is well below 3% when λ_M_ is less than 0.1 (solid square in **C**; corresponds to a *Drosophila* R1-R6 photoreceptor at 10^6^ photons/s). However, it approaches to 35% as λ_M_ increases to 1 (hollow square, corresponding to a species with 1000 microvilli stimulated at 10^6^ photons/s). This means that the *quantum-gain-nonlinearity* contributes differently to gain control, depending on the number of photons in the input and the number of microvilli in the photoreceptor's structure. It could potentially be a major contributor to the nonlinear output range compression, when a photoreceptor is under intense light stimulation (black dots in **D**), or if the cell has fewer photon detection units (green dots in **E**). λ_M_ is well below 0.1 for fly photoreceptors, as they have tens of thousands of microvilli to sample the available photons. Even in midday sunshine, quantum-gain factor is low (only ~3% for a *Drosophila* R1-R6 photoreceptor at 10^6^ photons/s; the solid square). Thus, *quantum-gain-nonlinearity* makes negligible contributions to *Drosophila* photoreceptors' gain control.

In general, there can be three cases for the multi-photon-hit-induced QB production: linear summation, sub-linear summation, and no summation (Figure 4). In linear summation, *x*-photons elicit a QB, in which size ($C_{x}^{QB}$) is equal to *x* times single photon induced QB size ($C_{1}^{QB}$). In the other extreme, there is no summation in QB production, and $C_{x}^{QB}$ equals to $C_{1}^{QB}$. More realistically, sublinear summation takes place in QB production, i.e., the resultant QB is bigger than a single photon response, but smaller than the sum of those produced independently. $C_{x}^{QB}$ is then always bounded between $C_{1}^{QB}$ and $xC_{1}^{QB}$ (gray areas in Figures 4B,C):

(5) $$\begin{array}{cc} C_{1}^{QB}\leq C_{x}^{QB}<xC_{1}^{QB}\;\begin{array}{cc} , & x\geq 1. \end{array} & \;(5) \end{array}$$

Substitute Equation (5) to Equation (4), *C*_out_/*L*_in_ is lower bounded in the case of no-summation:

(6) $$\begin{array}{cc} \frac{C_{\text{out}}}{L_{\text{in}}}\geq \frac{N_{\text{QB}}C_{1}^{QB}}{N_{\text{ph}}}=\frac{N_{\text{A}}C_{1}^{QB}}{N_{\text{ph}}}, & \;(6) \end{array}$$

where *N*_QB_ is the total number of QBs, and *N*_A_ is the total number of activated microvilli. As multi-photon-hits induce only one QB, it follows that *N*_QB_ is equal to *N*_A_, which is less or equal to *N*_ph_.

From Equation (6), the photoreceptor's input-output gain for a discrete light pulse ($\frac{C_{out}}{L_{\text{in}}}$) is lower bounded by $\frac{N_{\text{A}}C_{1}^{QB}}{N_{\text{ph}}}$, whose value is determined by two factors, *N*_*A*_/*N*_ph_ and $C_{1}^{QB}$, respectively. *N*_A_/*N*_ph_ is the ratio between the amount of activated microvilli (*N*_A_) and the number of incoming photons (*N*_ph_) at a particular time bin (Δ*t*, 1 ms here). This is the gain caused by the microvillar sampling process alone (Figure 1B), where the only constraint is that multi-photon-hits induce just one quantum bump, irrespective of its shape. On the other hand, $C_{1}^{QB}$ is determined by the bump shape, which can be viewed as an amplification of a single photon by the second-messenger signaling pathway in the phototransduction cascade.

*C*_out_/*L*_in_ is upper bounded in the case of linear summation:

(7) $$\begin{array}{cc} \frac{C_{\text{out}}}{L_{\text{in}}}=\frac{\sum_{x=1}^{x_{n}}N_{x}C_{x}^{QB}}{N_{\text{ph}}}<\frac{C_{1}^{QB}\sum_{x=1}^{x_{n}}xN_{x}}{N_{\text{ph}}}\approx \frac{N_{\text{ph}}C_{1}^{QB}}{N_{\text{ph}}}=C_{1}^{QB}. & \;(7) \end{array}$$

To focus on the role of photon sampling on gain control, we define $\frac{C_{out}}{L_{in}C_{1}^{QB}}$ as the normalized gain. This has an upper bound value of 1 in linear summation, and a lower bound value of *N*_*A*_/*N*_ph_ in no summation. More often, sublinear summation occurs in QB production, and the normalized gain is bounded between *N*_*A*_/*N*_ph_ and 1 (Figure 4B).

### Gain control induced by a sampling process

Markedly, the lower bound of the system's normalized gain (*N*_*A*_/*N*_ph_) is not influenced by the QB shape or any subsequent phototransduction processes, but is purely determined by photon sampling (the counted hits). From Section Realization of Random Photon Absorption Model *(RandPAM)*, we obtain a theoretical formula for *N*_A_/*N*_ph_ (random variable x is not limited by *x*_n_):

(8) $$\begin{array}{cc} \frac{N_{A}}{N_{\text{ph}}}=\frac{N_{u}(\sum_{x\geq 1}P(x))}{N_{\text{ph}}}=\frac{1-P(0)}{\lambda_{\text{M}}}=\frac{1-e^{-\lambda_{\text{M}}}}{\lambda_{\text{M}}}, & \;(8) \end{array}$$

where the function$\left(1-e^{-\lambda_{\text{M}}}\right)/\lambda_{\text{M}}$ is monotonic decreasing with respect to λ_M_ > 0. Owing to multi-photon-hit probabilities, photon sampling causes a nonlinear function of λ_M_, which varies with the light intensity and the number of photoreceptor microvilli. For a given light intensity, λ_M_ → 0 with more microvilli, and the normalized gain (*N*_A_/*N*_ph_) approaches 1, realizing linear photon sampling (Figure 4D). On the other hand, *N*_A_/*N*_ph_ decreases with increasing λ_*M*_. For a cell with a fixed number of microvilli, the normalized gain (*N*_A_/*N*_ph_) decreases with increasing light intensity, providing an elementary form of gain control in light adaptation (Figure 4E).

### Multi-photon hit rates

To quantify multi-photon induced *quantum-gain-nonlinearity* effects, we next calculate the likelihood for simultaneous multi-photon-hits to the same microvillus, and then estimate how the results depend upon the amount of microvilli.

**Figure 5:**
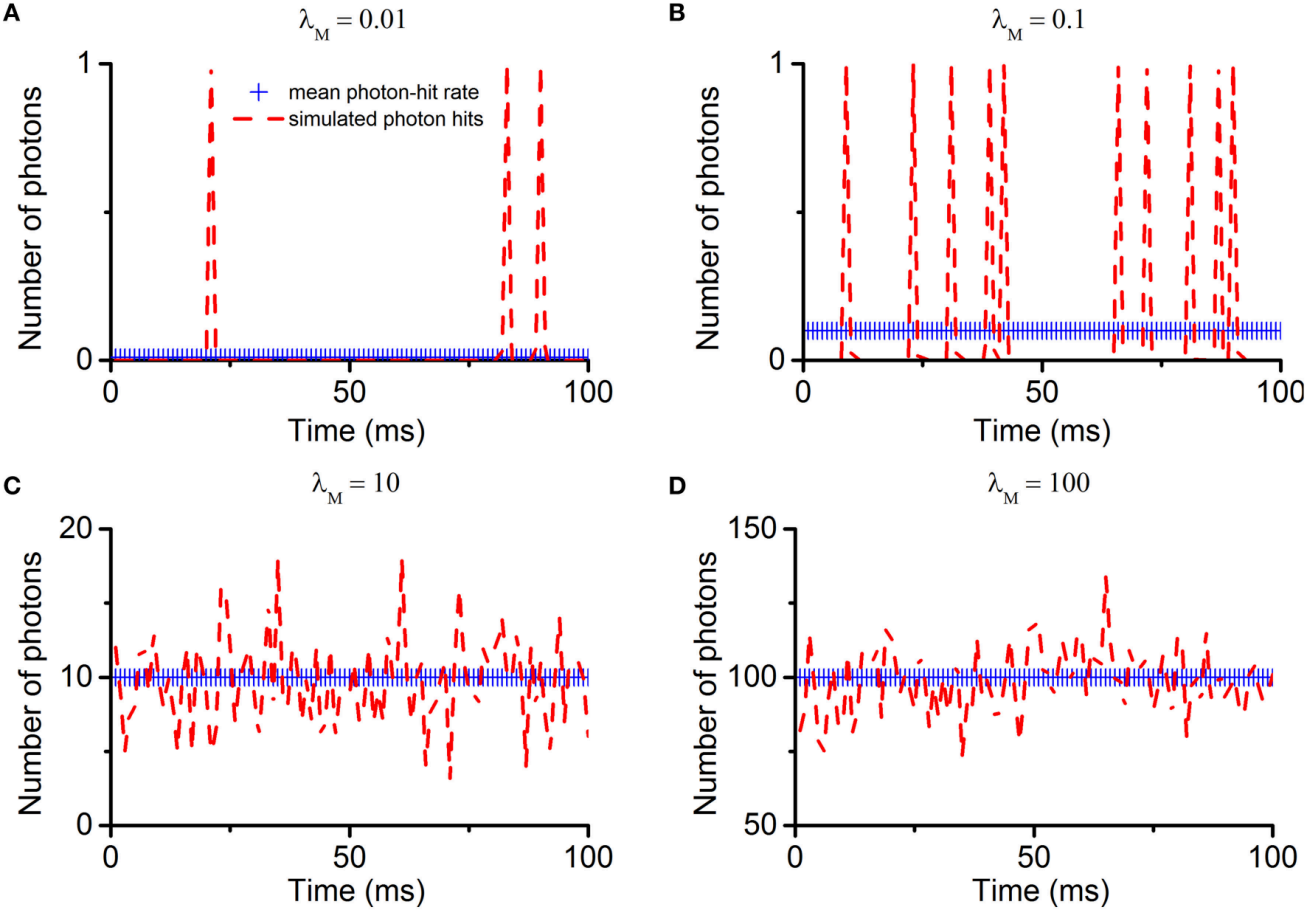
**Simulation of Poisson photon absorptions/hit to one microvillus**. Under the assumptions listed in Section Light Absorption by Poisson Process, photon absorptions (hits) to one microvillus follow the Poisson distribution. The photon-hit rate is the average number of absorptions in one microvillus within one time-bin ($\lambda_{{}^{\text{M}}}=N_{\text{ph}}/N_{\text{u}}$). The four sub-figures show the simulated Poisson photon-hits with different rate parameters. **(A)** λ_M_ = 0.01; **(B)** λ_M_ = 0.1; **(C)** λ_M_ = 10; **(D)** λ_M_ = 100. Notably, when λ_M_ is less than 1, discrete photon-hits are detected over time **(A,B)**. The smaller the λ_M_, the fewer photon-hits there are in a fixed time interval. But at any particular moment, the probability of multi-photon-hits is less than 1. When λ_M_ is greater than 1, photon-hits fluctuate around the mean **(C,D)**. Under such conditions, the multi-photon-hit probabilities increase with λ_M_, approximating 100% as λ_M_ approaches 5. As the *quantum-gain-nonlinearity* only affects phototransduction at the multi-photon-hit moments, its contribution should depend directly upon the photon hit rate.

Following the Poisson distribution, the multi-photon-hit probabilities depend directly upon the photon arrival rate. To illustrate this, we first simulate the photon arrivals to one microvillus. The key parameter is $\lambda_{{}^{\text{M}}}=N_{\text{ph}}/N_{\text{u}}$, which is the average number of photon-hits to each microvillus over one time-bin. The results are shown in Figure 5. When λ_M_ is less than 1, discrete photon-hits are detected over time (Figures 5A,B). The smaller the λ_M_, the fewer photon-hits there are in a fixed time interval. When λ_M_ is greater than 10, photon-hits fluctuate around the mean (Figures 5C,D). Under such conditions, the probabilities of multi-photon-hits are already approximating 100%.

The percentage of multi-photon-hits (*P*_M_) can be calculated by Equation (9):

(9) $$\begin{array}{cc} P_{\text{M}}=\frac{P(x>1)}{P(x\geq 1)}=\frac{1-P(0)-P(1)}{1-P(0)}=1-\frac{\lambda_{\text{M}}}{e^{-\lambda_{\text{M}}}-1}. & \;(9) \end{array}$$

Clearly, *P*_M_ increases with λ_M_, which is proportional to incoming photon rate and is in reciprocal relationship to the number of microvilli. So, multi-photon-hit-induced *quantum-gain-nonlinearity* should play a larger role with fewer microvilli, particularly at bright daylight, when multi-photon-hits are far more probable. With vast amount of microvilli, most of them experience only single-photon-hits and the *quantum-gain-nonlinearity* influences little the summed dynamics. Table 1 lists the multi-photon-hit percentages for the specified *N*_*ph*_ and *N*_*u*_ pairs. For example, a *Drosophila* R1-R6 photoreceptor has 30,000 microvilli. In dim and crepuscular conditions (*N*_ph_ = 10 and *N*_ph_ = 100, respectively), the percentage of multi-photon-hits on its microvilli is less than 0.17% (bold values in Table 1); thus, the *quantum-gain-nonlinearity* would have negligible effects on encoding. Even at very bright light levels (*N*_ph_ = 1000, corresponding to a bright midday: 10^6^ photons/s), *P*_M_ is still less than 2%.

**Table 1:** **Percentage of N photon-hits**.

***N*_ph_ photons/ms**	**Number of microvilli in the cell structure: *N*_u_**					
	**300 (%)**	**1500 (%)**	**6 k (%)**	**15 k (%)**	**30 k (%)**	**90 k (%)**
10	1.66	0.33	0.08	0.03	**0.02**	0.01
100	15.69	3.29	0.83	0.33	**0.17**	0.06
1 k	87.57	29.64	8.10	3.30	1.66	0.55
10 k	100	99.15	61.18	29.66	15.74	5.45
100 k	100	100	99.99	99.15	87.67	45.47

### Quantum-gain-nonlinearity effects on gain control

In this section, we quantify how quantum-gain-nonlinearity contributes to gain control (gray area in Figure 4). From Equation (7), the normalized gain upper bound ($\frac{C_{\text{out}}}{L_{in}C_{1}^{QB}}$) is 1, which corresponds to the case when every photon excites a QB, and these sum up linearly. Hence, the normalized linear gain is 1. Now, we define a quantum-gain factor (*Q*_*g*_), which is the difference between the normalized linear gain and the actual normalized gain ($\frac{C_{\text{out}}}{L_{in}C_{1}^{QB}}$), to quantify the contribution of the *quantum-gain-nonlinearity* on gain control:

(10) $$\begin{array}{cc} \begin{array}{l} Q_{g}=\frac{C_{1}^{QB}-\frac{C_{\text{out}}}{L_{\text{in}}}}{C_{1}^{QB}}=\frac{C_{1}^{QB}-\frac{\sum_{x=1}^{x_{n}}N_{x}C_{x}^{QB}}{N_{\text{ph}}}}{C_{1}^{QB}}\\ \;=1-\frac{\sum_{x=1}^{x_{n}}N_{\text{u}}C_{x}^{QB}P(x)}{N_{\text{ph}}C_{1}^{QB}}=1-P(0)-\frac{\sum_{x=2}^{x_{n}}\frac{C_{x}^{QB}}{C_{1}^{QB}}P(x)}{\lambda_{\text{M}}}, \end{array} & \;(10) \end{array}$$

where *P*(*x*) is the photon absorption probability calculated by Equation (2). $C_{x}^{QB}$ is the *x*-photon elicited QB charge (the area of the QB waveform).

Using $C_{x}^{QB}$values obtained from Figure 3, the black lines in Figure 4 illustrate how *Q*_*g*_ increases with λ_M_. It could rise to 35% as λ_M_ approaches 1 (indicated by the hollow square). This means that the *quantum-gain-nonlinearity* could potentially be a major contributor to the nonlinear range compression, when the cell has fewer photon sampling units. For fly photoreceptors, which have tens of thousands of microvilli to sample the available photons, λ_M_ is well below 0.1. Even in midday sunshine, *Q*_*g*_ is low (Figure 4C, the solid square). So *quantum-gain-nonlinearity* makes negligible contributions to *Drosophila* photoreceptors' gain control.

### Realistic photon sequence input to drive phototransduction cascades of individual microvilli

**Figure 6:**
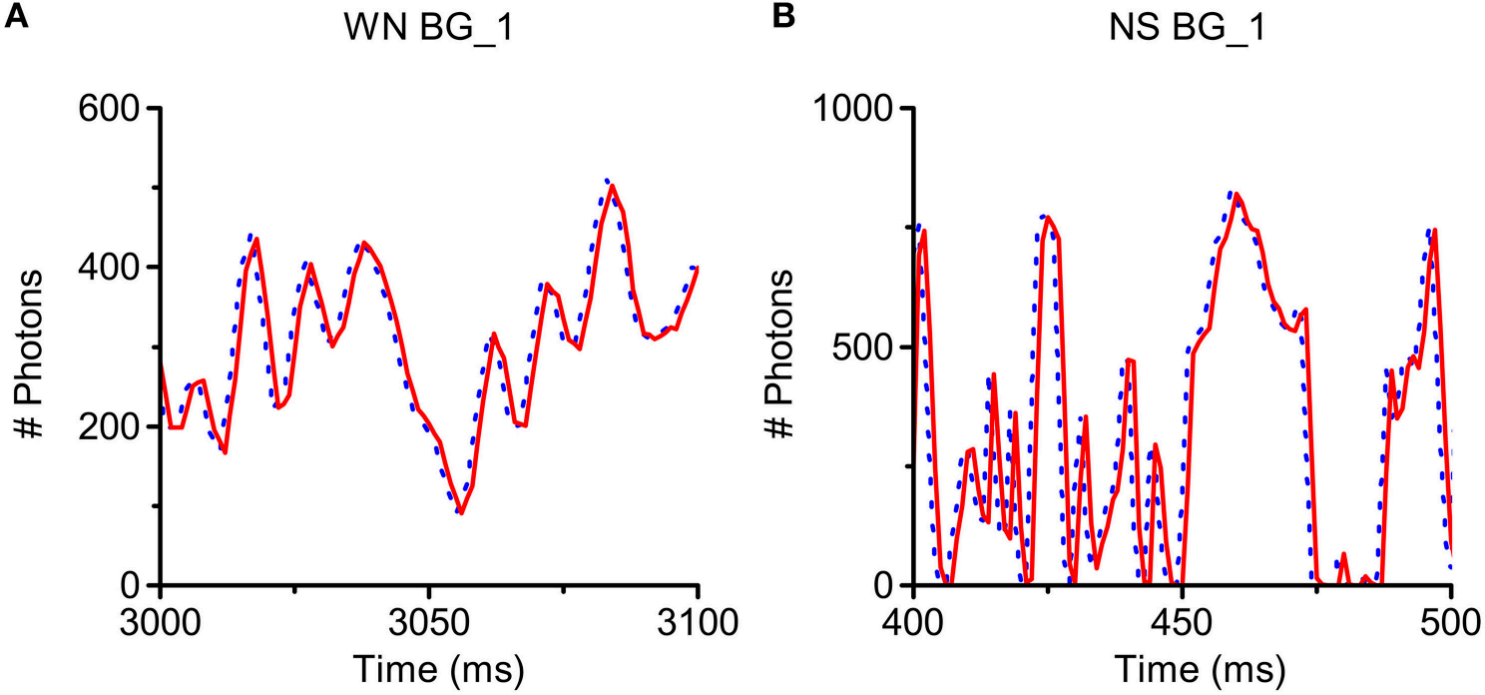
**Simulation of Poisson light absorption model for different light intensity time series**. *RandPAM* can be used to simulate photon arrival process of a fly photoreceptor to any light intensity time series with different statistical properties. Here, we test it with two light sequences, a 20 Hz band-limited Gaussian white noise (WN) and a naturalistic stimulus (NS), whose power spectra follows 1/*f* statistics (*f*: temporal frequency). The light intensity is given as photons/ms. *RandPAM* takes the photons in the stimuli and distributes them into a given number of microvilli (sampling units). The photon-hits of all microvilli must add up to the original input, as the model's underlying assumption is that all the photons become absorbed. The model produces identical light patterns as the input **(A,B)**. **(A)** Simulation of WN pattern at BG_1 (light level of 10^5^ photons/s); **(B)** Simulation of NS pattern at BG_1 (light level of 10^5^ photons/s). In each sub-figure, the dotted line (blue) is the light input and the solid line (red) is the simulation result. Because these curves overlap perfectly, the blue curves are right-shifted by 1 ms.

*RandPAM* can be used to simulate the photon arrival process of a fly photoreceptor to any light intensity time series with different statistical properties. Here, we test it with two light sequences: a 20 Hz band-limited Gaussian white noise and a naturalistic light intensity time series. These have band-limited and 1/*f* power spectra, respectively. As a standard practice, the light intensity is given as photons/ms. *RandPAM* takes the photons in the stimuli and distributes them pseudo-randomly into a given number of microvilli (sampling units). Each microvillus may thus be bombarded by a discrete sequence of photons over time. The photon-hits of all microvilli must add up to the input level, as the model's underlying assumption is that all the photons become absorbed. Figures 6A,B show that the model produces identical light patterns as the input.

Because *RandPAM* can take any light intensity time series and map it as photon-hit-sequences over the tested microvillus population, it is essentially a spread of excitation model that distributes the photon arrivals to individual microvilli, driving their separate phototransduction cascades (Song et al., 2012; Song and Juusola, 2014). Thus, *RandPAM* realizes a photoreceptor's essential input-output mapping and makes it possible to study the related encoding problems.

## Discussion

In this report, we gave a detailed account of *RandPAM*, which is specifically designed for studying photon sampling in fly photoreceptors. We explained how *RandPAM* distributes incoming photons over a large population of microvilli (30,000 in *Drosophila* photoreceptor Boschek, 1971; Hardie and Postma, 2008), and provided its assumptions and derivations.

We used *RandPAM* to answer specific questions about the early gain control in photon sampling. If photoreceptors were purely photon counters, counting every single photon that hits them, their limited output range would readily saturate in direct sunlight, which can flux in tens of millions of photons per photoreceptor's receptive field per second (Warrant and Mcintyre, 1992). To combat this burden, it has been suggested that the sublinear summation in early transduction reactions may reduce the (QB size)/photon gain at instances of multi-photon-hits (Pumir et al., 2008). As this multi-photon-hit induced gain control originates in QB production, we defined it as *quantum-gain-nonlinearity*.

By reducing *quantum-gain-nonlinearity* to the time-resolution limit, we showed how gain control already emerges from photon sampling alone. This means that adaptation begins even before phototransduction reactions shape the quantum bump waveform. The implementation is just photon sampling by a finite population, with the only constraint being that multi-photon-hits reduce to a single event of sublinear amplitude.

Importantly, *RandPAM* provided quantification of how the *quantum-gain-nonlinearity* affects phototransduction, as a function of the photon count in the light input and the photoreceptor's microvillus (sampling unit) count. We showed that its contribution is marginal (≤ 1%) for fly photoreceptors with many thousands of microvilli sampling incoming photons. On the contrary, for small insect photoreceptors with a limited number of microvilli, the probability of simultaneous multi-photon-hits to a single microvillus would be higher, and therefore these coincidences may affect it encoding more.

These results clarify that diurnal fly photoreceptors have enough microvilli (30,000–100,000) to maintain high photon-hit rates without *quantum-bump nonlinearity* much affecting their encoding. Aligned with our previous studies (Song et al., 2012; Song and Juusola, 2014), we conclude that it is not the number of microvilli, but refractoriness and speed of their phototransduction cascades that ultimately limit the temporal signaling precision and the information transfer rate of fly photoreceptors. This realization means that there are likely further evolutionary trade-offs, such as the metabolic costs and space restrictions for maintaining large microvilli populations that optimize their numbers to the flies' lifestyle and habitat requirements.

Finally, we speculate that *quantum-gain-nonlinearity* may affect light adaptation in cilliary photoreceptors more, especially when they face a bright daylight environment. This is because cilliary photoreceptors normally have fewer photon-capturing-units, for example, the outer segment of a toad rod contains about 2000 pancake-like disks (Sjostrand, 1953; Pugh and Lamb, 2000). If these disks were to act as photon sampling and phototransduciton units, the theoretical results in Sections Random Photon Absorption Model *(RandPAM)* and Results may also apply. The modest amount of disks could make a cilliary photoreceptor's encoding more prone to the *quantum-gain-nonlinearity* at bright light. Thus, there could be important differences in the way quantum gain control is implemented in rhabdomeric and ciliary photoreceptors.

## Author contributions

ZS, MJ designed the study. ZS, YZ constructed the model and performed the analysis. ZS drafted the manuscript with all authors editing it.

### Conflict of interest statement

The authors declare that the research was conducted in the absence of any commercial or financial relationships that could be construed as a potential conflict of interest.

## Appendix: Simulating a fly photoreceptor's macroscopic nonlinear voltage output to dynamic light input.

We recently developed a full biophysical fly photoreceptor model that can transduce any light intensity time series input into macroscopic output, the light-induced current (LIC) (Song et al., 2012). A fly photoreceptor's LIC response to a temporally varying photon flux is highly dynamic and nonlinear. The full model simulates such responses by adding up semi-independent quantum bumps. For example, Figures 4C,D in Song et al. (2012) show the simulated photoreceptor voltage output to dim and bright naturalistic light intensity time series. The model was validated by the close correspondence between the simulations and intracellular recordings at different light conditions.

These simulations were performed by the procedure described in Figure 1 of this paper. Semi-independent stochastic phototransduction cascades of 30,000 microvilli were simulated, and all their quantum bumps summed up the macroscopic responses. RandPAM provided the photon input to the microvilli.

We have further shown (Song et al., 2012) that the nonlinear temporal light adaptation dynamics depend upon:

- Light input statistics
- Number of microvilli (sampling units)
- Stochastic refractory period in each microvillus
  ◦ The success of transducing a photon into a quantum bump depends upon refractoriness of its phototransduction reactions. This means that a microvillus cannot respond to the next photons until its phototransduction reactions have recovered from the previous photon absorption, which takes about 50-300 ms
- Stochastic latency distribution (the time delay between photon arrivals to the trigged quantum bump)
- Bump waveform adaptation over time
- Voltage feedback from the plasma membrane

The details how these mechanisms jointly modulate photoreceptor output dynamics to different light intensity time series input can be found in our previous publications (Song et al., 2012; Juusola et al., 2015).
